# The Discovery of Weddellamycin, a Tricyclic Polyene Macrolactam Antibiotic from an Antarctic Deep-Sea-Derived *Streptomyces* sp. DSS69, by Heterologous Expression

**DOI:** 10.3390/md22040189

**Published:** 2024-04-21

**Authors:** Lu Chen, Kai Liu, Jiali Hong, Zhanzhao Cui, Weijun He, Yemin Wang, Zixin Deng, Meifeng Tao

**Affiliations:** 1 State Key Laboratory of Microbial Metabolism, School of Life Sciences and Biotechnology, Shanghai Jiao Tong University, Shanghai 200240, China; chenlu0310@sina.com (L.C.); kailiucn@163.com (K.L.); hongjlbio@163.com (J.H.); cuizhanzhao@sjtu.edu.cn (Z.C.); weijunhe@sjtu.edu.cn (W.H.); wangyemin@sjtu.edu.cn (Y.W.); zxdeng@sjtu.edu.cn (Z.D.); 2 Haihe Laboratory of Synthetic Biology, Tianjin 300308, China; 3 Tianjin Institute of Industrial Biotechnology, Chinese Academy of Sciences, Tianjin 300308, China

**Keywords:** *Streptomyces* sp. DSS69, genome mining, heterologous expression, polyene macrolactam, production increase, antibacterial activity, cytotoxicity

## Abstract

Polyene macrolactams are a special group of natural products with great diversity, unique structural features, and a wide range of biological activities. Herein, a cryptic gene cluster for the biosynthesis of putative macrolactams was disclosed from a sponge-associated bacterium, *Streptomyces* sp. DSS69, by genome mining. Cloning and heterologous expression of the whole biosynthetic gene cluster led to the discovery of weddellamycin, a polyene macrolactam bearing a 23/5/6 ring skeleton. A negative regulator, WdlO, and two positive regulators, WdlA and WdlB, involved in the regulation of weddellamycin production were unraveled. The fermentation titer of weddellamycin was significantly improved by overexpression of *wdlA* and *wdlB* and deletion of *wdlO*. Notably, weddellamycin showed remarkable antibacterial activity against various Gram-positive bacteria including MRSA, with MIC values of 0.10–0.83 μg/mL, and antifungal activity against *Candida albicans*, with an MIC value of 3.33 μg/mL. Weddellamycin also displayed cytotoxicity against several cancer cell lines, with IC_50_ values ranging from 2.07 to 11.50 µM.

## 1. Introduction

Polyene macrolactams (PMLs) are a class of natural products featured by a 16–34-membered lactam ring bearing two isolated/separated polyene fragments, which often undergo intramolecular cyclization to afford complex polycyclic scaffolds [1]. The structural diversity of PMLs is largely attributed to the unique features of their biosynthetic pathways, including β-amino acids as starting units and transannular cyclization reactions, among others [2]. As a result of their structural diversity, PMLs display a wide spectrum of bioactivities such as antiviral (kenalactams A-E) [3], antibacterial (BE-14106 and auroramycin) [4,5], antifungal (streptolactams A and C) [6], and antitumor (cyclamenol E and FW05328-1) [7,8] activities.

Actinobacteria from terrestrial and marine environments are predominately producers of natural PMLs, such as *Streptomyces*, *Micromonospora*, and *Nocardiopsis* [9,10,11,12]. Particularly, *Streptomyces* comprise more than half of all reported PMLs. Recent bioinformatics analyses unveiled a wide distribution of cryptic biosynthetic gene clusters (BGCs) for putative PMLs [12]. Indeed, genome mining has been successfully used to identify new PMLs [13,14,15]. Due to the difficulties in the genetic manipulation of many bacterial strains, heterologous expression of selected gene clusters has become an efficient strategy for activating silent gene clusters, mining the genomes of new natural products, and characterizing biosynthetic pathways [16].

In the current work, we report the following: (i) a cryptic PML BGC (*wdl*), identified through genome sequence analysis of a sponge-associated *Streptomyces* sp. DSS69; (ii) a new PML (compound **1**), produced by the *wdl* BGC via its heterologous expression in *Streptomyces lividans* GX28; (iii) the functions of four cluster-situated regulatory genes in the *wdl* BGC and the application of these genes in enhancing the production of compound **1**; and (iv) the promising bioactivities of **1** against *Candida albicans*, Gram-positive bacteria including MRSA, and cancer cells.

## 2. Results

### 2.1. Secondary Metabolic Potential of Streptomyces sp. DSS69

*Streptomyces* sp. DSS69 was isolated from a marine sponge sample collected from the Weddell Sea in Antarctica [17]. Whole genome sequencing revealed that this strain contains a linear chromosome of 7,704,811 bps with a G+C content of 71.7% (Figure S1). Sequence alignment using 16S rDNA indicated that *Streptomyces* sp. DSS69 is most closely related to the *Streptomyces microflavus* strain NA06532 (100% identity) and the *Streptomyces fulvorobeus* strain DSM 41,455 (100% identity). Bioinformatics analysis using the online tool antiSMASH [18] predicted that the genome of *Streptomyces* sp. DSS69 contains 36 putative secondary metabolite BGCs, which totally occupy 1.36 Mb and 17.7% of the complete genome. These BGCs were predicted to be responsible for the biosynthesis of four polyketides (PKs), eight nonribosomal peptides (NRPs), three hybrid PK-NRPs, six ribosomally synthesized and post-translationally modified peptides (RiPPs), and fifteen others (Table S1).

**Figure 1:**
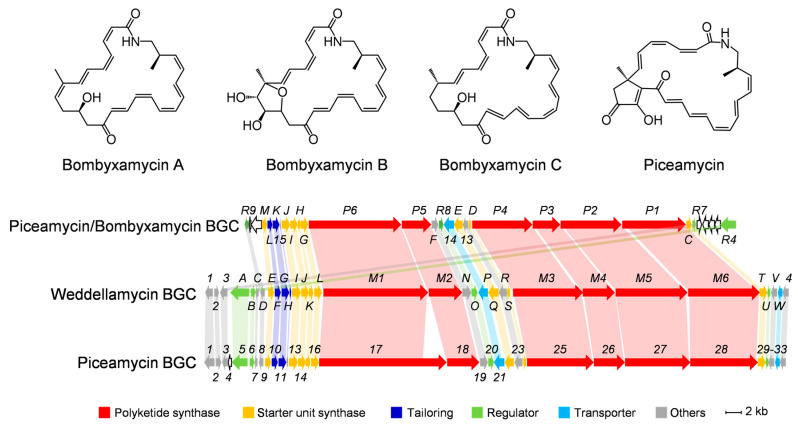
Comparison of the *wdl* BGC with the bombyxamycin and piceamycin BGC of *Streptomyces* sp. SD53 and the piceamycin BGC of *Streptomyces* sp. AmelAP-1. Shaded bars between BGCs identify homologous genes between the three BGCs. The hollow arrows indicate no homologous genes among the three BGCs.

BGC15 of *Streptomyces* sp. DSS69 is a putative type I polyketide synthase (PKS) BGC (named as *wdl* BGC hereafter). It displays high similarity to several BGCs for the biosynthesis of polyene macrolactam antibiotics, particularly the bombyxamycins (*bom*) from *Streptomyces* sp. SD53 [19,20] and the piceamycin BGC from *Streptomyces* sp. AmeAP-1 [21] in terms of gene composition and gene organization (Figure 1). The *wdl* BGC encodes thirty-two gene products, including six PKSs, eight enzymes involved in the biosynthesis of a β-amino acid as the start unit of the polyketide chain, a P450 monooxygenase, and four regulatory genes (Table S2).

However, our extensive efforts failed to identify bombyxamycin or piceamycin from the fermentation culture of *Streptomyces* sp. DSS69, implying that the *wdl* BGC was either silent in *Streptomyces* sp. DSS69 or coding for an unknown compound.

### 2.2. Isolation and Characterization of Weddellamycin (1) Produced by Heterologous Expression of the wdl BGC

**Figure 2:**
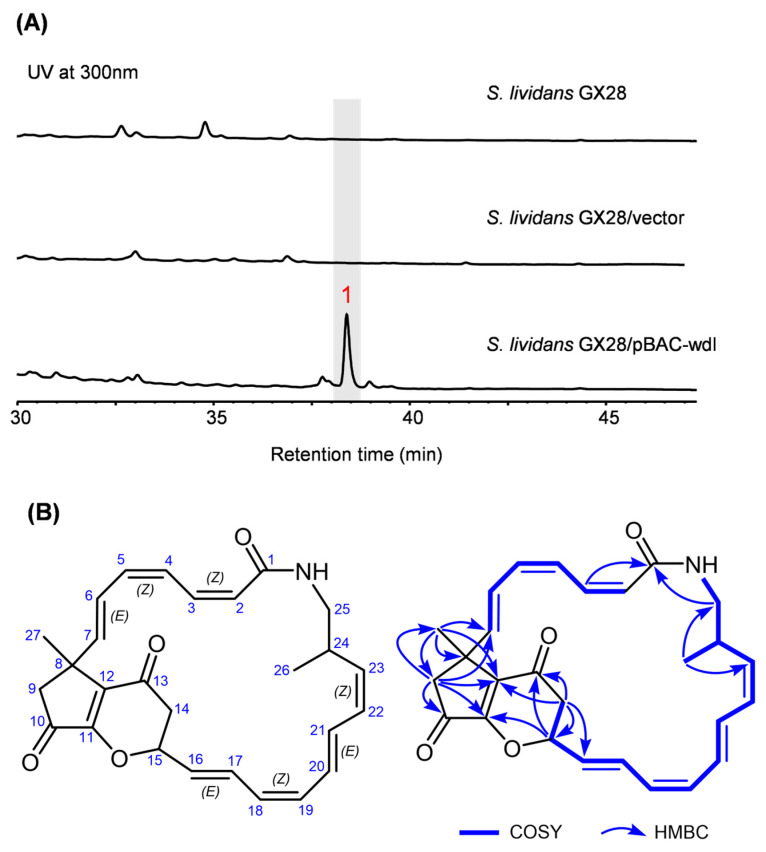
Identification of weddellamycin (**1**) from *S. lividans* GX28/pBAC-wdl. (**A**) HPLC analysis of the crude extract of *S. lividans* GX28/pBAC-wdl. The vector is the cloning vector pMSBBAC1. (**B**) Planar structure, key COSY, and HMBC of compound **1**.

A bacterial artificial chromosome (BAC) genomic library was constructed to capture the wdl BGC for heterologous expression in a surrogate host. A BAC clone covering the entire wdl BGC, named pBAC-wdl, was obtained by PCR screening using four pairs of primers matching to the predicted left end (orf1), the right end (orf4), and two PKS genes (wdlM1 and wdlM5) in the BGC (Table S3). The plasmid pBAC-wdl was conjugated into *Streptomyces lividans* GX28, a productive heterologous expression host [22]. The empty BAC vector pMSBBAC1 [23] was also introduced into *S. lividans* GX28 as a negative control. High-performance liquid chromatography (HPLC) analysis of the resulting strains revealed a new peak (**1**) from the crude acetonitrile extract of *S. lividans* GX28/pBAC-wdl (Figure 2A). Compound **1** was isolated from a 10 L fermented culture of *S. lividans* GX28/pBAC-wdl.

Compound **1**, obtained as a yellow powder, possesses a molecular formula of C_27_H_29_NO_4_, determined by high-resolution electrospray ionization mass spectrometry—HR-ESI-MS—(m/z 432.2166 [M+H]^+^, calcd. 432.2175, Figure S2), implying 14 degrees of unsaturation. The infrared data (Figure S3) showed a strong and broad band at the left end of the spectrum, at 3422 cm^−^^1^ for the N-H stretch, and a band in the middle of the spectrum at 1631 cm^−^^1^ for the C=O stretch, indicating the existence of amide bond(s). The ^1^H NMR spectrum (Table 1 and Figures S4–S8) in DMSO-*d_6_* of **1** showed one exchangeable proton signal at δ_H_ 7.65 (1H, dd, J = 7.5, 4.3 Hz, NH), fourteen coupling splitting olefinic protons in the range of 5.0‒7.0 ppm, one oxygen-bearing methine proton at δ_H_ 5.61 (1H, br dd, J = 6.4, 3.7 Hz, H-15), and seven aliphatic protons in the range of 2.4‒3.2 ppm, as well as one singlet methyl and one doublet methyl in the high field region. The ^13^C NMR spectrum (Table 1 and Figures S4–S8) exhibited a total of twenty-seven carbon signals, including two conjugated ketone carbonyl carbons at δ_C_ 202.1 (s, C-10) and 191.1 (s, C-13), one conjugated amide carbonyl carbon at δ_C_ 165.6 (s, C-1), sixteen olefinic carbons due to eight double bond groups, and one oxygenated methine carbon at δ_C_ 78.0 (d, C-15), as well as seven aliphatic carbons (1C, 1CH, 3CH_2_, and 2CH_3_) in the high field region. Three carbonyl functionalities and eight double bond groups contributed 11 degrees of unsaturation; therefore, there must be three rings in the structure. Considering its biological source, the above NMR features allowed us to speculate that structure **1** is probably a macrolactam polyketide. The NMR data for compound **1** are similar to those of piceamycin [20], originally discovered from *Streptomyces* sp. GB4-2 [24] and later also found to be produced by *Streptomyces* sp. SD53 [20] and *Streptomyces* sp. AmelAP-1 [21].

Careful analysis of the ^1^H and ^1^H-COSY correlations (Figure 2B) and the characteristic coupling constants of the double bond groups confirmed the existence of two sets of conjugated polyenic coupled systems. Further comparison of **1** and piceamycin revealed that the main differences were that **1** had one less double bond group and one more ring than piceamycin. Furthermore, the HMBC correlations (Figure 2B) from the oxygenated methine proton to C-11 [δ_C_ 158.8 (s)] and C-13 [δ_C_ 191.1 (s)], and from H-14 to C-12, C-13, C-15, and C-16 confirmed that the ∆^14^ was dihydrogenated and a pyran ring was formed via a new ether bond (C-11—O—C-15). The resulting planar structure, especially the cyclopenta[b]pyran-4,7-dione moiety and the configurations of double bond groups, was further verified by the 2D NMR analysis. Regrettably, the NOESY experiment failed to determine the relative configuration of the rigid 6,5-fused bicyclic part because of the planarity of the conjugated diketone moiety.

Compound **1** is named as weddellamycin. The structure of weddellamycin (**1**) is similar to piceamycin, except for a newly emerged six-membered dihydropyran-4-one ring fused to the 2-cyclopentenone ring of piceamycin, which leads to the formation of an unusual tricyclic skeleton for **1**. Thus, compound **1** represents a unique 23/5/6-tricyclic polyene macrolactam, highlighted by its substituted tetrahydrocyclopenta[b]pyran-4,7-dione moiety.

### 2.3. Proposed Biosynthetic Pathway of Weddellamycin

**Figure 3:**
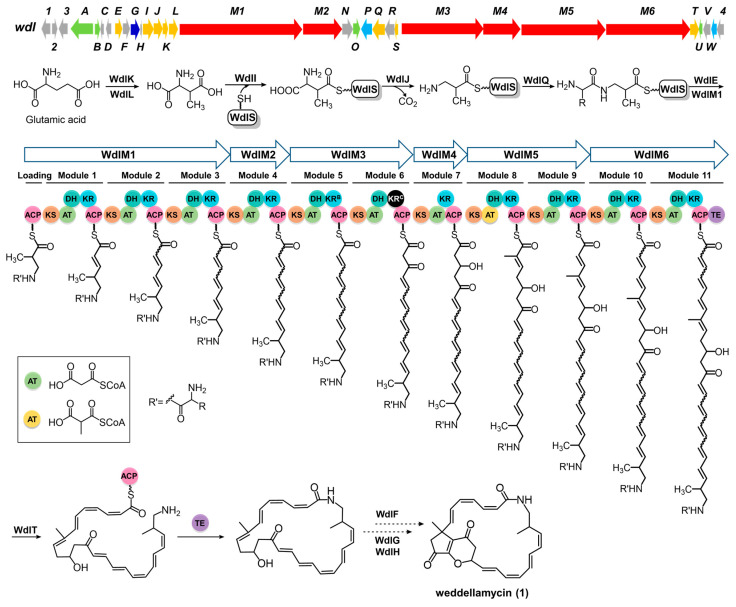
The proposed biosynthetic pathway of weddellamycin (**1**). ACP, acyl carrier protein (pink); KS, ketosynthase (orange); KR, ketoreductase (blue); AT, acyltransferase; DH, dehydratase (dark green); TE, thioesterase (purple). The colored AT domains are derived from malonyl-CoA (light green) and methylmalonyl-CoA (yellow). The black KR domain indicates inactivation according to prediction.

The high gene-to-gene similarity between wdl BGC and the piceamycin/bombyxamycin BGCs suggests that weddellamycin is produced as illustrated in Figure 3, via a biosynthetic pathway analogous to those of bombyxamycin and piceamycin [19,20] except for the final cyclization step(s).

An *N*-acyl group-protected β-amino acid starter unit is proposed to be synthesized from L-glutamic acid by a set of enzymes, i.e., glutamate mutase WdlK and WdlL, acyl carrier protein WdlS, decarboxylase WdlJ, and two ATP-dependent ligases, WdlI and WdlQ. Subsequently, an ACP S-acyltransferase WdlE helps with the loading of the N-acyl group-protected β-amino acid starter unit to WdlM1, the first member of the PKS assembly line.

The PKS assembly line for the core macrolactam ring of weddellamycin is constituted by WdlM1-M6, which contains a loading module and 11 elongation modules. Bioinformatic analysis revealed that the acyltransferase (AT) domain in module 8 is methylmalonyl-CoA-specific, as it contains a YASH motif. This is congruent with the structure of weddellamycin, which has a methyl group at position 8. Other AT domains contain the HAFH motif, suggesting a selectivity for malonyl-CoA (Figure S9A) [25,26]. The ketoreductase (KR) domain in module 6 was predicted to be redox-inactive and part of the C1 type due to its lack of the catalytic tyrosine (“Y motif”), and that explained the ketone group at C-13 of the PKS(ACP)-tethered long-chain precursor (Figure S9B) [27,28]. The dehydratase (DH) domain is absent in module 7, which is consistent with the hydroxy group at C-11 [29]. The presence of DH and KR domains in the other modules gives rise to double bonds on the polyketide chain. After the formation of the polyene chain, the terminal protective acyl group of the chain is removed by the L-amino acid amidase WdlT (a homologue of BomC) prior to macrocyclization by the thioesterase domain of WdlM6.

Eventually, three tailoring enzymes—WdlG (putative cytochrome P450), WdlH (putative ferredoxin), and WdlF (putative isomerase/epimerase)—took the role in the post-PKS modifications, yielding the final product, weddellamycin (**1**) (Figure 3). Consistently, compound **1** was not produced in the gene-deletion mutants *S. lividans* GX28/pBAC-*ΔwdlF*, *ΔwdlG*, and *ΔwdlH* (Figure S10).

### 2.4. Enhancing the Production of Weddellamycin

Since compound **1**, like other PLMs, is not very stable [30,31] and the production of compound **1** in the heterologous expression host was very low (0.29 mg/L), it was difficult to accumulate sufficient pure substrate for bioactivity tests. We turned to the *wdl* BGC-situated regulatory genes to enhance the production. The *wdl* gene cluster encodes one LuxR family regulator (WdlA), two TetR family regulators (WdlB and WdlO), and one GntR regulator (WdlU). Protein sequence analysis revealed that WdlA is homologous to the well-characterized positive cluster-situated regulators AveR (identity/similarity: 40%/53%) from *Streptomyces avermitilis* [32] and SlnR (identity/similarity: 46%/59%) from *Streptomyces albus* [33]. WdlB is homologous to ArtX (identity/similarity: 30%/50%), a positive regulator from *Streptomyces aurantiacus*, JA4570 [34]. WdlO is homologous to a negative regulator, SAV_576 (identity/similarity: 31%/51%), from *Streptomyces avermitilis* [35]. WdlU is homologous to IndYR, a positive regulator from *Streptomyces globisporus* (identity/similarity: 88%/92%) [36].

**Figure 4:**
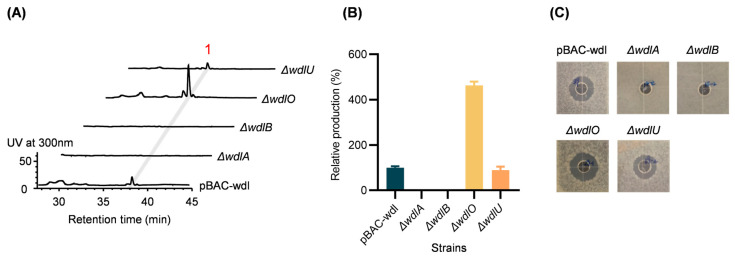
Effects of *wdlA*, *wdlB*, *wdlO*, and *wdlU* gene deletion on weddellamycin biosynthesis. (**A**) The HPLC profiles of *S. lividans* GX28/pBAC-wdl and the gene deletion mutants ∆wdlA, ∆wdlB, ∆wdlO, and ∆wdlU. (**B**) Quantitative analysis of weddellamycin production in *S. lividans* GX28/pBAC-wdl and the mutants. The production of weddellamycin in *S. lividans* GX28/pBAC-wdl is present as 100%. (**C**) The biological activity against Bacillus altitudinis of *S. lividans* GX28/pBAC-wdl and the four gene deletion mutants. Crude extract (20 μL) was added to the central wells in the agar plates premixed with *B. altitudinis* as an indicator. Biological activity of weddellamycin (**1**) was indicated by the zones of growth inhibition after 24 h of incubation at 37 °C.

To study the roles of these regulators in the production of weddellamycin, *wdlA*, *wdlB*, *wdlO*, and *wdlU* were deleted, respectively, from pBAC-wdl using λ RED-mediated PCR targeting (Figure S10). The four resultant gene deletion plasmids were transferred into *S. lividans* GX28 via conjugation, yielding four gene deletion mutants. HPLC analysis of the fermentation extracts showed that the *wdlA* or *wdlB* deletions abolished production and the *wdlO* deletion increased production more than threefold, whereas the *wdlU* deletion did not affect the production of weddellamycin significantly (Figure 4A,B). Biological activity assays of the extracts of the mutants produced consistent results (Figure 4C). These results suggest that WdlA and WdlB play positive roles, WdlO plays a negative role, and WdlU plays an undetectable role in the regulation of weddellamycin biosynthesis.

**Figure 5:**
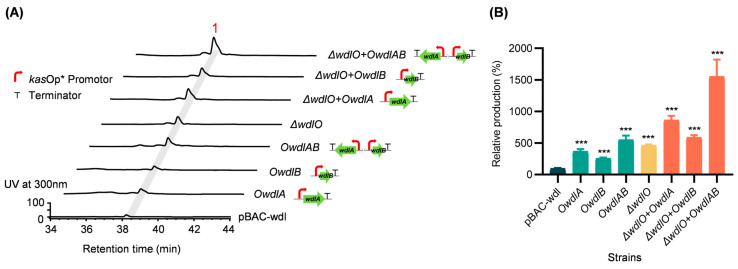
Effects of the overexpression of *wdlA* and/or *wdlB* on the production of weddellamycin in *S. lividans* GX28/pBAC-wdl and GX28/pBAC-*ΔwdlO*. (**A**) HPLC analysis of the production of weddellamycin (**1**) in *S. lividans* GX28/pBAC-wdl, the *ΔwdlO* mutant, and their overexpression derivatives. (**B**) Quantitative analysis of the weddellamycin produced in the overexpression strains. The production of weddellamycin in *S. lividans* GX28/pBAC-wdl is present as 100%. * *p* < 0.05, *** *p* < 0.001, as determined by the two-tailed Student’s t-test. *OwdlAB*, *wdlA*, and *wdlB* were overexpressed. *OwdlA*, *wdlA* were overexpressed. *OwdlB*, *wdlB* were overexpressed.

To confirm the regulatory roles of *wdlA* and *wdlB* and to further increase the production of weddellamycin, *wdlA* and/or *wdlB* were overexpressed under the control of *kasO*p*, a strong constitutive synthetic promoter, via integrative constructs (Figure S11) in the *S. lividans* strains GX28/pBAC-wdl and GX28/pBAC-*ΔwdlO*. As anticipated, the fermentation titers of weddellamycin in all the overexpression mutants were substantially increased compared to those of the parent strain GX28/pBAC-wdl, as indicated by the HPLC peaks (Figure 5). Quantitative analysis revealed that the production of weddellamycin in GX28/*ΔwdlO*+*OwdlAB*, in which *wdlA* and *wdlB* were overexpressed, improved most significantly, by 15 folds (*p* = 7.04 × 10^−^^4^, 4.43 mg/L,) relative to *S. lividans* GX28/pBAC-wdl.

### 2.5. Biological Activities

The antibacterial activity of compound **1** was assessed against seven Gram-positive bacteria, a fungal strain, and a Gram-negative bacterium. As shown in Table 2, compound **1** exhibited potent activity against all the Gram-positive bacterial and fungal strains, with minimum inhibitory concentration (MIC) values in the range of 0.10 to 3.33 μg/mL. However, there was no effective activity of **1** against the Gram-negative bacterium *E. coli*.

The in vitro cytotoxicity of compound **1** against human leukemia HL-60, human hepatoma HepG2, human glioblastoma U-87MG, and human colon cancer HCT 116 was also measured. The results of these assays revealed that compound **1** exhibits potent cytotoxicity against these cell lines, with IC_50_ values between 2.07 and 11.50 μM (Table 3).

## 3. Materials and Methods

### 3.1. General Experimental Procedures

The UV spectra were recorded using a Thermo Fisher EV300 UV-vis spectrophotometer (Waltham, MA, USA). The optical rotations were determined with a JASCO P-2000 digital polarimeter (Mary’s Court Easton, MD, USA). IR spectra were recorded on a Thermo Fisher Nicolet 6700 spectrometer, peaks are reported in cm^−1^. The 1D and 2D NMR spectra were recorded on a Bruker Avance III 600 MHz spectrometer (Billerica, MA, USA), and HRESIMS spectra were recorded with a Waters Acquity UPLC I-class coupled with a Vion IMS QTOF (Milford, MA, USA). ECD spectra were obtained using a JASCO J-1500 spectrometer. Flash chromatography was carried out using the BUCHI Pure C-805 flash system (New Castle, DE, USA) with an Airs Science Flash C18-M column (20–35 μm, 100 Å, 90 g). Preparative RP-HPLC and HPLC analyses were performed with the Waters Prep 150 LC system and an Agilent 1260 HPLC system, using an Agilent ZORBAX SB C-18 column (5 µm, 9.4 × 250 nm) and an Agilent ZORBAX SB C-18 column (5 µm, 4.6 × 250 nm), respectively. Column chromatography (CC) was carried out using a DIAION HP20 column (Mitsubishi Chemical Co., Tokyo, Japan) and Sephadex LH-20 gel (GE Healthcare, Uppsala, Sweden). All solvents employed for CC were of analytical grade (Shanghai Chemical Reagents Co., Ltd., Shanghai, China); those for HPLC and HRESIMS were of UV-HPLC- and UPLC/LC-MS-gradient grade (ANPEL Laboratory Technologies [Shanghai] Inc., Shanghai, China), respectively.

### 3.2. Strains, Plasmids, Primers, and Culture Conditions

The strains, plasmids, and primers used in this study are listed in Tables S1 and S2. *Streptomyces* sp. DSS69 was isolated from a sponge sample collected from the Weddell Sea (200–4800 m deep) in Antarctica in 2005–2006 by the Xue and Zhang Group [17]. A 16S rDNA analysis was used to determine the taxonomic identity via alignment with sequences from the GenBank database using BLAST (nucleotide sequence comparison). *Streptomyces* sp. DSS69 was preserved in 20% glycerol aqueous solution at −80 °C in the State Key Laboratory of Microbial Metabolism, School of Life Sciences and Biotechnology, Shanghai Jiao Tong University, China.

The *S. lividans* TK24-derived strain GX28 [22] was used as a heterologous expression host. *E. coli* DH10B was used for routine DNA cloning. *E. coli* ET12567/pUB307 [37] was used to facilitate the intergeneric triparental conjugation. *E. coli* BW25113/pIJ790 [38] was used for λ Red-mediated PCR targeting to construct gene deletion mutants. *E. coli* DH5α/BT340 [38] was used for the construction of in-frame deletion mutants using flippase recombination enzyme (FLP)-mediated site-specific recombination. pMSBBAC1 [23] containing the origin of transfer (*oriT*), the *φC31* integrase gene, the integrating *attP* site, and an apramycin resistance gene was used as the BAC vector for constructing the BAC library. pMSBBAC1-derived plasmids (BAC clones and related gene disruption mutants) were mobilized and integrated into the chromosome of *Streptomyces* spp. at the *attB_φC31_* attachment site. pMS82 [39], bearing φBT1-derived integrase gene *attP^φBT1^*, was used as the backbone for gene cloning and overexpression.

Luria-Bertani (LB) medium was used for all *E. coli* growth. Mannitol soya flour (MS) medium [22] (20 g soybean flour, 20 g mannitol, and 20 g agar per liter of water) was used for *Streptomyces* and its derivatives’ growth, sporulation, and conjugation. *Streptomyces* mycelia were inoculated in TSBY medium (103 g sucrose, 5 g yeast extract, and 30 g tryptone soy broth per liter of water) for genomic DNA extraction. The solid medium for fermentation and isolation of the compound was R5a medium (100 g sucrose, 0.25 g K_2_SO_4_, 10.12 g MgCl_2_·6H_2_O, 5 g yeast extract, 0.1 g casamino acid, 10 g D-glucose, 21 g MOPS, and 20 g agar per liter water) with 2 mL of trace element solution added per liter (40 μg NaOH, 20 μg ZnCl_2_, 20 μg FeCl_3_·6H_2_O, 10 μg MnCl_2_, and 10 μg (NH_4_)_6_Mo_7_O_24_·4H_2_O per liter water) [40]. All cultures for *Streptomyces* were incubated at 30 °C. Apramycin (50 µg/mL), erythromycin (300 µg/mL), hygromycin B (50 µg/mL), nalidixic acid (25 µg/mL), and trimethoprim (50 µg/mL) were used when necessary.

### 3.3. Genome Sequencing and Bioinformatic Analysis

*Streptomyces* sp. DSS69 was incubated in TSBY liquid medium (50 mL) in 250 mL Erlenmeyer flasks at 30 °C for 48 h at 220 rpm. Subsequently, the mycelia were collected by centrifugation at 4000 rpm for 10 min at 4 °C, washed three times with phosphate-buffered saline (PBS), and stored at −80°C. Genomic DNA extraction and whole-genome sequencing were performed by Shanghai Personalbio Technology Co., Ltd. (Shanghai, China), using the PacBio Sequel and Illumina Miseq platforms. Biosynthetic gene clusters in the genome of *Streptomyces* sp. DSS69 were analyzed and assessed using antiSMASH. MIBiG and 2ndFind were used to predict and analyze the functions of ORFs. UniProt was used for the protein blast. The whole genome of *Streptomyces* sp. DSS69 has been deposited at GenBank under accession number CP142147.

### 3.4. BAC Library Construction and Screening

The mycelia of *Streptomyces* sp. DSS69 was obtained and prepared as described in Section 3.3. The extraction of genomic DNA and the construction of the BAC library was completed by Eight Star Bio-tech Co., Ltd., Hubei, China [23]. High-molecular weight DNA fragments were prepared by being partially digested with *Sau*3AI and ligated to *Sau*3AI-digested pMSBBAC1. The ligation mixture was electroporated into *E. coli* DH10B-competent cells, resulting in a genomic BAC library including approximately 2000 clones that were stored in 24 96-well plates at *−*80 °C.

BAC -wdl, which contains the wdl gene cluster, was identified by PCR using primers 15-1-F/R to 15-4-F/R (Table S2). These primers are located at both ends and in the middle of the predicted biosynthetic gene clusters. By amplifying these four fragments, the plasmid pBAC-wdl was identified and obtained.

### 3.5. Heterologous Expression, Fermentation, and Isolation

Plasmid pBAC-wdl was introduced into *S. lividans* GX28—termed *S. lividans* GX28/pBAC-wdl—via triparental mating, using *E. coli* ET12567/pUB307 as a helper strain. The exconjugants containing intact BAC clones were verified by PCR with primers 15-1-F/R to 15-4-F/R. The correct independent transconjugant was cultured on MS plates at 30 °C for 4 to 6 days for sporulation. Then the Streptomyces spores were collected and incubated on R5a solid medium (40 mL per 9 cm petri dish) at 30 °C for 7 days for large-scale fermentation. The fermented culture was pressed through a fine sieve and extracted thrice with acetonitrile (ACN) to yield a crude extract.

The *S. lividans* GX28/pBAC-wdl organic extract from 10 L of fermentation medium was subjected to HP20 using a step gradient elution with methanol (MeOH) and H_2_O (MeOH/H_2_O: 0, 50, 100%, *v*/*v*). The 100% MeOH fraction (1.5 g) was dissolved in 90% MeOH/H_2_O and extracted with hexane three times. After removing the hexane part, the remainder was concentrated and subjected to a flash C18-M column with a linear gradient of elution buffer (30–100% MeOH-H_2_O, 30 min) and a Sephadex LH-20 column eluted with MeOH to remove most non-target compounds. Then the fraction was separated by semipreparative HPLC elution with 68% MeOH/H_2_O (2 mL/min, t_R_ = 16.9 min, 300 nm) and purified with 47% ACN/H_2_O to afford compound **1** (1.6 mg, 2 mL/min, t_R_ = 31.5 min, 300 nm). In order to reduce the degradation of the compound, strains were cultured in the dark, and the purification process was carried out under low-light conditions. The sample collection vials were covered by foil. Finally, the compound was retrieved by a freeze dryer.

Compound **1**, a yellowish powder, comprises [α]^D^_25_ −62.2 (c 0.02, DMSO-d_6_); UV/Vis (DMSO): λmax (log ɛ) = 227.0 (0.323), 256.0 (0.390), 295.0 (0.658) nm; IR (neat, cm^−1^) 3422, 2927, 1631, 1453, 1384, 1248, 1123, 1056, and 1000. ^1^H NMR (600 MHz, DMSO- d_6_) and ^13^C NMR (150 MHz, DMSO- d_6_) data are shown in Table 1; HRESIMS m/z 432.2166 [M + H]^+^ (calcd for C_27_H_30_NO_4_^+^, 432.2175).

### 3.6. Construction of Gene Deletion and Overexpression Mutants

The λ-RED-mediated PCR-targeting method was used to delete specific regulator genes (Figure S11). The primers designed for gene-specific deactivation can be found in Table S2. As an example, the process for deleting gene *wdlA* is explained here. First, pBAC-wdl was introduced into *E. coli* BW25113/pIJ790. Then, an erythromycin resistance gene cassette (*eryB*) from pJTU6722, which was flanked by FLP recognition sites, was amplified using primers ΔwdlA-F and ΔwdlA-R. The purified PCR product of the erythromycin resistance gene cassette was transformed into *E. coli* BW25113/pIJ790/pBAC-wdl by electroporation to replace gene *wdlA*. Next, the gene replacement construct was introduced into *E. coli* BT340 and cultured at 42 °C to remove the *eryB* cassette through FLP-mediated excision, leaving an 81-bp scar. This resulted in the creation of the plasmid pCL01. The plasmid was confirmed by PCR analysis with primers ΔwdlA-YZ-F and ΔwdlA-YZ-R, and by DNA sequencing (Figure S11). Plasmids pCL02 to pCL07, which were single-gene deletions of *wdlB*, *wdlF*, *wdlG*, *wdlH*, *wdlO*, or *wdlU*, were constructed following similar procedures. The resulting mutated BACs were then introduced into *S. lividans* GX28, yielding strains *S. lividans* GX28/*ΔwdlA*, *S. lividans* GX28/*ΔwdlB*, *S. lividans* GX28/*ΔwdlF*, *S. lividans* GX28/*ΔwdlG*, *S. lividans* GX28/*ΔwdlH*, *S. lividans* GX28/*ΔwdlO*, and *S. lividans* GX28/*ΔwdlU*.

To overexpress positive regulator genes, plasmids pCL08, pCL09, and pCL10 were constructed, carrying genes under the control of the widely used Streptomyces strong promoter *kasO*p* (Figure S11). Fragments of *kasO*p*-*wdlA*-ter or *kasO*p*-*wdlB*-ter-1 were amplified from the BAC -wdl, using the primers *kasO*p*-*wdlA*-ter-F/R or *kasO*p*-*wdlB*-ter-1-F/R. Then the cassette was inserted into the pMS82 vector (digested with *Not*I and *Spe*I) using the ClonExpress One Step Cloning Kit to form plasmids pCL08 and pCL09, which are responsible for overexpressing the *wdlA* and *wdlB* genes, respectively. The plasmid pCL10 was constructed by inserting a cassette *kasO*p*-*wdlB*-ter-2, amplified from pBAC-wdl with the primer *kasO*p*-*wdlB*-ter-2-F/R, into the plasmid pCL08 using the method described above to overexpress both the *wdlA* and *wdlB* genes. The plasmid pCL08 was linearized by *Avr*II and *Hin*dIII digestion. The identities of all plasmids were confirmed by PCR analysis and DNA sequencing. The verified plasmids were transferred to *S. lividans* GX28/pBAC-wdl and *S. lividans* GX28/*ΔwdlO*, respectively, by using *E. coli* ET12567/pUB307-mediated triparental conjugation, yielding strains *S. lividans* GX28/*OwdlA*, *S. lividans* GX28/*OwdlB*, and *S. lividans* GX28/*OwdlAB*, or strains *S. lividans* GX28/*ΔwdlO*+*OwdlA*, *S. lividans* GX28/*ΔwdlO*+*OwdlB*, and *S. lividans* GX28/*ΔwdlO*+*OwdlAB*.

### 3.7. Metabolic Analysis

The fermentation of the strain *S. lividans* GX28/-wdl and its derivatives was performed as described in Section 3.5. Each type of conjugate had three replicates, each with three plates for fermentation.

All plates were cut and soaked in an equal volume of ACN (40 mL each), and incubated overnight. The organic layer was centrifuged at 13,500 rpm for 15 min and analyzed by an Agilent 1260 HPLC system with an Agilent Zorbax SB-C18 column (5 µm, 4.6 × 250 nm), using H_2_O (solvent A) and 100% MeOH (solvent B) as the mobile phase. For HPLC analysis, the elution system of MeOH/H_2_O (0–40 min, 5–100% MeOH; 40–50 min, 100% MeOH; and 50.01–60 min, 5% MeOH) was carried out at a flow rate of 0.5 mL/min. The detection wavelength was 300 nm.

### 3.8. Antibacterial and Antifungal Activity Assay

Compound **1** was evaluated for in vitro bioactivity against some bacteria and fungi. Compound **1** dissolved in DMSO was prepared by sequential 2-fold serial dilution in a 96-well plate in MH medium at the final concentrations of 0.052, 0.10, 0.21, 0.42, 0.83, 1.67, 3.33, 6.67, 13.33, and 26.67 μg/mL. Ampicillin was used as a control with a maximum test concentration of 100 μg/mL. After incubation, the plates were examined and MIC values were calculated.

The cytotoxicity of compound **1** was determined by CCK-8 assay [41] with four human cancer cell lines HL-60, HCT 116, HepG2, and U-87MG. Each cell line was exposed to the tested compound at concentrations of 40, 20, 10, 5, 1, 0.1, and 0.01 µM in triplicate. After 48 h, cell viability was measured by a CCK-8 Kit according to the manufacturer’s instructions. DOX was used as a positive control.

## 4. Conclusions

The genome of a sponge-associated bacterium, *Streptomyces* sp. DSS69, was completely sequenced, from which a cryptic polyene macrolactam (PML) biosynthetic gene cluster, *wdl*, was identified via genome mining. A strategy combining the BAC library and heterologous expression enabled the activation of the *wdl* BGC, leading to the identification of a new PML, weddellamycin (**1**), which harbors a unique 23/5/6-tricyclic macrolactam scaffold. A biosynthetic pathway of compound **1** was proposed based on the comparison with homologous gene clusters. However, the precise biochemistry and enzymology involved in the formation of the tetrahydrocyclopenta[*b*]pyran-4,7-dione bicyclic system and the stereochemistry selectivity remain elusive and await further investigation.

Three cluster-situated regulatory genes were demonstrated to modulate the production of weddellamycin (**1**). Overexpression of *wdlA* and *wdlB* and deletion of *wdlO*, either alone or in combination, led to a remarkable enhancement in the production of compound **1**, with a maximum increase of 15.5 folds. Additionally, compound **1** was shown to be active against a fungal pathogen—*Candida albicans*—and Gram-positive bacteria, including MRSA.

These findings not only provide a specific new polyene macrolactam congener for the development of new anti-infectious agents, but they also create a foundation for future combinatorial biosynthesis to improve the availability of PMLs and other related natural product-based drug leads.

## Figures and Tables

**Table 1:** ^1^H and ^13^C NMR spectroscopic data for compound **1** in DMSO-*d_6_*.

No.	^1^H NMR	^13^C NMR	No.	^1^H NMR	^13^C NMR
1		165.6 (C)	15	5.61 (1H, br dd, 6.4, 3.7)	78.0 (CH)
2	5.49 (1H, d, 11.4)	123.5 (CH)	16	5.76 (1H, dd, 15.5, 3.7)	129.4 (CH)
3	6.49 (1H, dd, 11.8, 11.4)	131.8 (CH) *	17	6.43 (1H, dd, 15.5, 11.2)	129.0 (CH)
4	6.89 (1H, dd, 11.8, 10.8)	124.1 (CH)	18	5.95 (1H, dd, 11.2, 10.8)	126.9 (CH)
5	5.95 (1H, dd, 11.2, 10.8)	133.6 (CH)	19	6.08 (1H, dd, 11.6, 10.8)	132.5 (CH)
6	6.37 (1H, dd, 15.3, 11.2)	122.8 (CH)	20	6.28 (1H, dd, 14.8, 11.6)	126.5 (CH)
7	5.75 (1H, d, 15.3)	143.2 (CH)	21	6.54 (1H, dd, 14.8, 11.2)	131.7 (CH) *
8		39.8 (C) **	22	6.00 (1H, dd, 11.2, 11.0)	129.4 (CH)
9	2.45 (1H, d, 19.2) 2.55 (1H, d, 19.2)	49.5 (CH_2_)	23	5.13 (1H, dd, 11.0, 9.2)	136.9 (CH)
10		202.1 (C)	24	2.92 (1H, m)	32.5 (CH)
11		158.8 (C)	25	2.96 (1H, ddd, 12.5, 10.1, 7.5) 3.05 (1H, ddd, 12.5, 4.3, 3.7)	44.4 (CH_2_)
12		138.4 (C)	26	0.94 (3H, d, 6.3)	18.0 (CH_3_)
13		191.1 (C)	27	1.42 (3H, s)	24.1 (CH_3_)
14	2.90 (1H, br d, 17.6) 3.15 (1H, dd, 17.6, 6.4)	38.6 (CH_2_)	NH	7.65 (1H, dd, 7.5, 4.3)	

* Interchangeable. ** Overlapped with the solvent signal. See Supplementary Materials for the NMR spectra.

**Table 2:** Antimicrobial activities of compound **1** (MIC, μg/mL).

Strains	1	Ampicillin
*Staphylococcus aureus* ATCC25923	0.21	0.20
MRSA	0.10	50
MRSE	0.21	>100
*Enterococcus faecalis* ATCC29212	0.83	>100
*Micrococcus luteus* ATCC4698	0.21	0.39
*Bacillus altitudinis* 41KF2b	0.21	0.20
*Listeria monocytogenes* ATCC BAA-679	0.10	3.12
*Candida albicans*	3.33	>100
*Escherichia coli* DH10B	>27	100

**Table 3:** Cytotoxic activities of compound **1** (IC_50_, μM).

Cell Line	1	DOX
HL-60	4.93 ± 0.26	0.51 ± 0.02
HepG2	11.50 ± 0.14	0.19 ± 0.01
HCT 116	2.07 ± 0.04	0.07 ± 0.01
U-87MG	8.76 ± 0.12	0.09 ± 0.01

## Data Availability

Data are contained within the article or Supplementary Materials.

## Supplementary Materials

The following supporting information can be downloaded at: https://www.mdpi.com/article/10.3390/md22040189/s1, Table S1—antiSMASH-predicted BGCs for *Streptomyces* sp. DSS69; Table S2—Predicted function of the genes from the weddellamycin BGC; Table S3—Primers used in this study; Table S4—Strains and plasmids used and constructed in this study; Figure S1—The complete genome and features of S*treptomyces* sp. DSS69; Figures S2–S8—HR-ESI-MS, IR and 1D and 2D NMR spectra of compound **1**; Figure S9—Multiple sequence alignment of AT domains and KR domains; Figure S10—Disruptions of weddellamycin biosynthetic genes via PCR targeting and PCR verification; Figure S11—Schematic maps of overexpressed plasmids and PCR verification [22,23,37,38,39,42,43].
