# Supplementary material for: The Discovery of Weddellamycin, a Tricyclic Polyene Macrolactam Antibiotic from an Antarctic Deep-Sea-Derived Streptomyces sp. DSS69, by Heterologous Expression
Source: Mar Drugs. 2024 Apr 21;22(4):189. doi: 10.3390/md22040189 (PMC11051340; doi:10.3390/md22040189)
Supplement: Supplementary file 1 [file marinedrugs-22-00189-s001.zip › marinedrugs-2856588-supplementary.pdf]

# The Discovery of Weddellamycin, a Tricyclic Polyene Macrolactam Antibiotic from an Antarctic Deep-Sea-Derived *Streptomyces* sp. DSS69, by Heterologous Expression

**Lu Chen[1], Kai Liu[1], Jiali Hong[1], Zhanzhao Cui[1], Weijun He[1], Yemin Wang[1], Zixin Deng[1, 2, 3] and Meifeng Tao[1, 2, *]**

[1] State Key Laboratory of Microbial Metabolism, School of Life Sciences and Biotechnology, Shanghai Jiao Tong University, Shanghai, 200240, China;

[2] Haihe Laboratory of Synthetic Biology, Tianjin 300308, China;

[3] Tianjin Institute of Industrial Biotechnology, Chinese Academy of Sciences, Tianjin 300308, China;

chenlu0310@sina.com (L.C.); kailiucn@163.com (K.L.); hongjlbio@163.com (J.H.); cuizhanzhao@sjtu.edu.cn (Z.C.); weijunhe@sjtu.edu.cn (W.H.); wangyemin@sjtu.edu.cn (Y.W.); zxdeng@sjtu.edu.cn (Z.D.)

* Correspondence: tao_meifeng@sjtu.edu.cn (M.T.)

## Supporting Information Table of Contents

| Entry | Contents | Pages |
|---|---|---|
| 1. | **Table S1.** antiSMASH-predicted BGCs for *Streptomyces* sp. DSS69. | 2 |
| 2. | **Table S2.** Predicted function of the genes from the weddellamycin BGC. | 3 |
| 3. | **Table S3.** Primers used in this study. | 5 |
| 4. | **Table S4.** Strains and plasmids used and constructed in this study. | 7 |
| 5. | **Figure S1.** The complete genome and features of *Streptomyces* sp. DSS69. | 9 |
| 6. | **Figure S2-S8.** HR-ESI-MS, IR and 1D and 2D NMR spectra of compound **1**. | 10-13 |
| 7. | **Figure S9.** Multiple sequence alignment of AT domains and KR domains. | 14 |
| 8. | **Figure S10.** Disruptions of weddellamycin biosynthetic genes via PCR-targeting and PCR verification. | 15 |
| 9. | **Figure S11.** Schematic maps of overexpression plasmids and PCR verification. | 16 |

**Table S1.** antiSMASH-predicted BGCs for *Streptomyces* sp. *DSS69*.

| BGCs | Position | | Type | Most similar known cluster/Similarity |
|---|---|---|---|---|
| | from | to | | |
| Cluster 1 | 115,769 | 126,656 | butyrolactone | coelimycin P1 / 16% |
| Cluster 2 | 155,631 | 177,513 | terpene | geosmin / 100% |
| Cluster 3 | 217,831 | 270,224 | NRPS | griseobactin / 100% |
| Cluster 4 | 294,564 | 350,623 | NRPS | coelichelin / 81% |
| Cluster 5 | 360,973 | 399,099 | T3PKS | lasalocid / 9% |
| Cluster 6 | 680,259 | 722,829 | NRPS | diisonitrile antibiotic SF2768 / 66% |
| Cluster 7 | 1,020,062 | 1,039,917 | terpene | steffimycin D / 19% |
| Cluster 8 | 1,485,361 | 1,495,259 | ectoine | ectoine / 100% |
| Cluster 9 | 2,073,506 | 2,146,039 | T2PKS-oligosaccharide | chromomycin A3 / 100% |
| Cluster 10 | 2,438,892 | 2,469,166 | lanthipeptide | — |
| Cluster 11 | 2,524,339 | 2,534,794 | siderophore | desferrioxamin B / 100% |
| Cluster 12 | 2,883,003 | 2,916,534 | LAP-thiopeptide | — |
| Cluster 13 | 3,034,928 | 3,109,440 | NRPS-PKS-like | omnipeptin / 7% |
| Cluster 14 | 3,136,407 | 3,188,955 | NRPS | phosphonoglycans / 3% |
| Cluster 15 | 3,311,291 | 3,417,260 | T1PKS | bombyxamycin A / 50% |
| Cluster 16 | 3,426,050 | 3,479,268 | lanthipeptide-NRPS | albomycin delta2 / 10% |
| Cluster 17 | 3,669,942 | 3,697,519 | betalactone | divergolide / 6% |
| Cluster 18 | 4,115,741 | 4,136,103 | nucleoside | toyocamycin / 30% |
| Cluster 19 | 4,305,025 | 4,327,695 | lassopeptide | keywimysin / 100% |
| Cluster 20 | 5,107,055 | 5,129,670 | lanthipeptide | AmfS / 100% |
| Cluster 21 | 5,454,333 | 5,473,242 | terpene | — |
| Cluster 22 | 5,846,508 | 5,861,249 | siderophore | kanamycin / 20% |
| Cluster 23 | 6,176,336 | 6,186,306 | RiPP-like | — |
| Cluster 24 | 6,327,416 | 6,373,542 | NRPS | salinomycin / 14% |
| Cluster 25 | 6,426,691 | 6,477,940 | ectoine | kosinostatin / 13% |
| Cluster 26 | 6,786,620 | 6,812,714 | terpene | hopene / 69% |
| Cluster 27 | 6,864,977 | 6,914,966 | NRPS | a201a / 8% |
| Cluster 28 | 6,927,927 | 6,976,192 | TIPKS-NRPS | 10-epi-HSAF / 100% |
| Cluster 29 | 6,991,610 | 6,999,604 | RiPP-like | tetronasin / 3% |
| Cluster 30 | 7,091,171 | 7,144,941 | NRPS | thiocoraline / 21% |
| Cluster 31 | 7,222,132 | 7,231,365 | RiPP-like | streptamidine / 66% |
| Cluster 32 | 7,233,826 | 7,244,293 | melanin | istamycin / 4% |
| Cluster 33 | 7,315,596 | 7,356,648 | T3PKS | alkylresorcinol / 100% |
| Cluster 34 | 7,364,029 | 7,430,038 | NRPS | crochelin A / 12% |
| Cluster 35 | 7,514,850 | 7,540,412 | terpene | isorenieratene / 100% |
| Cluster 36 | 7,554,728 | 7,606,949 | T1PKS-NRPS-thiopeptide | lactazole / 33% |

**Table S2.** Predicted function of the genes from the weddellamycin BGC.

| No. | Gene | Size [bp](aa) | Putative function | Homolog | Identity [%] / Similarity [%] |
|---|---|---|---|---|---|
| 1 | *orf1* | 1533 (510) | alpha / beta hydrolase fold protein | (AAP03104.1) | 69 / 92 |
| 2 | *orf2* | 789 (262) | thioesterase | CmiR1 (BAO66517.1) | 71 / 98 |
| 3 | *orf3* | 1128 (375) | putative transcriptional regulator | BomR5 (QBL56177.1) | 61 / 55 |
| 4 | *wdlA* | 2745 (914) | LuxR family transcriptional regulator | BomR4 (QBL56174.1) | 62 / 100 |
| 5 | *wdlB* | 603 (200) | TetR family transcriptional regulator | MlaM (ACO94493.1) | 50 / 93 |
| 6 | *wdlC* | 270 (89) | unknown | (QBL56203.1) | 74 / 100 |
| 7 | *wdlD* | 759 (252) | type II thioesterase | BomR9 (QBL56202.1) | 72 / 97 |
| 8 | *wdlE* | 948 (315) | ACP S-malonyltransferase | BomM (QBL56199.1) | 73 / 100 |
| 9 | *wdlF* | 870 (289) | sugar phosphate isomerase / epimerase | BomL (QBL56198.1) | 66 / 99 |
| 10 | *wdlG* | 1233 (410) | cytochrome P450 | BomK (QBL56197.1) | 88 / 100 |
| 11 | *wdlH* | 195 (4) | ferredoxin | (QBL56196.1) | 88 / 100 |
| 12 | *wdlI* | 1659 (552) | long chain fatty acid CoA ligase | BomJ (QBL56195.1) | 78 / 92 |
| 13 | *wdlJ* | 1260 (419) | diaminopimelate decarboxylase | BomI (QBL56194.1) | 81 / 99 |
| 14 | *wdlK* | 501 (166) | glutamate mutase sigma subunit 2 | BomH (QBL56193.1) | 73 / 89 |
| 15 | *wdlL* | 1428 (475) | methylaspartate mutase | BomG (QBL56192.1) | 75 / 89 |
| 16 | *wdlM1* | 16398 (5465) | PKS | | |
| 17 | *wdlM2* | 5322 (1773) | PKS | | |
| 18 | *wdlN* | 1206 (401) | FAD-dependent oxidoreductase | BomF (QBL56190.1) | 74 / 99 |
| 19 | *wdlO* | 756 (251) | TetR family transcriptional regulator | BomR8 (QBL56189.1) | 80 / 100 |
| 20 | *wdlP* | 1656 (551) | transporter | (QBL56188.1) | 81 / 94 |
| 21 | *wdlQ* | 1602 (533) | long chain fatty acid CoA ligase | BomE (QBL56187.1) | 73 / 99 |
| 22 | *wdlR* | 1035 (344) | transposase | (QBL56186.1) | 54 / 91 |

(table to be continued)

(table continued)

| | | | | | |
|---|---|---|---|---|---|
| 23 | *wdlS* | 237 (78) | acyl carrier protein | BomD (QBL56185.1) | 76 / 100 |
| 24 | *wdlM3* | 10644 (3547) | PKS | | |
| 25 | *wdlM4* | 5019 (1672) | PKS | | |
| 26 | *wdlM5* | 10995 (3664) | PKS | | |
| 27 | *wdlM6* | 11376 (3788) | PKS | | |
| 28 | *wdlT* | 909 (302) | L-amino acid amidase | BomC (QBL56180.1) | 87 / 99 |
| 29 | *wdlU* | 405 (134) | GntR family transcriptional regulator | (MXG30154.1) | 99 / 99 |
| 30 | *wdlV* | 975 (324) | ABC transporter permease subunit | (WP_109164968.1) | 100 / 100 |
| 31 | *wdlW* | 945 (314) | putative ABC transporter ATP-binding protein | (ACB47083.1) | 44 / 89 |
| 32 | *orf4* | 684 (227) | phosphotransferase | (WP_109164969.1) | 99 / 99 |

**Table S3.** Primers used in this study.

| Primer | Sequence (5′→3′) | Uses |
|---|---|---|
| 15-1-F | CGGGGAGGGGGAGTCAGATG | Primers for BAC plasmid screening |
| 15-1-R | CAGGGCAACTTCTGGCTCG | |
| 15-2-F | CGTTCGCTGCGGGAGGTCATC | |
| 15-2-R | GCTTCCAGGGTGAGTTCCTC | |
| 15-3-F | GTGCAGATGACTGAGTCGGG | |
| 15-3-R | GCCGCTCCAGGACGAAGACG | |
| 15-4-F | TCAGGTGCACTTCTTGTCGT | |
| 15-4-R | GCTATCTCCAGGGGTACGCG | |
| *Δ wdlA*-F | TTGACTTCTTTGCATGTCCTTGACTGCTGTGGGCGGTC ATGTAGGCTGGAGCTGCTTC | Primers for disrupting *wdlA* |
| *Δ wdlA*-R | CGGAAGCAGGCCGGTCAGCAGGATCTGTCCAGTGA AGTGATTCCGGGGATCCGTCGACC | |
| *Δ wdlA*-YZ-F | TACTGTCAGTGGCGAAACGG | Primers for *ΔwdlA* construct verification |
| *Δ wdlA*-YZ-R | CCGGCCCACCGTACGTGAGT | |
| *Δ wdlB*-F | ACCCGGCGGGGACCACCCGCACCACGGTCCCGGCC GTCATGTAGGCTGGAGCTGCTTC | Primers for disrupting *wdlB* |
| *Δ wdlB*-R | GATGGCCGCCACGACCGCGAGACGAGGGGGTGTGC CGTGATTCCGGGGATCCGTCGACC | |
| *Δ wdlB*-YZ-F | GGTCACTAGTTGCACCGTGC | Primers for *ΔwdlB* construct verification |
| *Δ wdlB*-YZ-R | GCCCCCGAGAACACCCGTAG | |
| *Δ wdlF*-F | GGAGTTCGGTGGCGGTGAGGGGGGCTCGGTGAGGG CCTATGTAGGCTGGAGCTGCTTC | Primers for disrupting *wdlF* |
| *Δ wdlF*-R | GGTCGGCCGTGAACTGTCGAAGGCGAGAGGTACCCC ATGATTCCGGGGATCCGTCGACC | |
| *Δ wdlF*-YZ-F | GTTCGGTGGCGGTGAGGGGG | Primers for *ΔwdlF* construct verification |
| *Δ wdlF*-YZ-R | GGTCGGCCGTGAACTGTCGA | |
| *Δ wdlG*-F | CGACGATCACACGCATGGAACCGCTCCTTTACCAGG TCATGTAGGCTGGAGCTGCTTC | Primers for disrupting *wdlG* |
| *Δ wdlG*-R | CACCGAACTCCGCCAAAGAGCTGAAGAGAGGCCAC CATGATTCCGGGGATCCGTCGACC | |
| *Δ wdlG*-YZ-F | ACGATCACACGCATGGAACC | Primers for *ΔwdlG* construct verification |
| *Δ wdlG*-YZ-R | CGCCAAAGAGCTGAAGAGAG | |
| *Δ wdlH*-F | CGCCCGGTCGGTGGTCCGGGTGCGCGGGGGGCCGCG CTATGTAGGCTGGAGCTGCTTC | Primers for disrupting *wdlH* |
| *Δ wdlH*-R | CACGAACTCCCGGTGACCTGGTAAAGGAGCGGTTCC ATGATTCCGGGGATCCGTCGACC | |
| *Δ wdlH*-YZ-F | CGGTCGGTGGTCCGGGTG | Primers for *ΔwdlH* construct verification |
| *Δ wdlH*-YZ-R | GACCTGGTAAAGGAGCGGTT | |
| *Δ wdlO*-F | TCCGGGGCGGGGGACCAGCCCCCTTCGGCCTGTGTG TCATGTAGGCTGGAGCTGCTTC | Primers for disrupting *wdlO* |
| *Δ wdlO*-R | TACGCACGGCCCTGCATCCACGAAGGTGAGCACCTT ATGATTCCGGGGATCCGTCGACC | |
| *Δ wdlO*-YZ-F | CTGCCGGCAGTTCCTCGGTG | Primers for *ΔwdlO* construct verification |
| *Δ wdlO*-YZ-R | CTCGGCGACTACGAGACCAC | |

(table to be continued)

(table continued)

| | | |
|---|---|---|
| *Δ wdlU*-F | CTGACCCCGCGACCCACCCACGTACGGTCGGGTCCGTCATGTAGGCTGGAGCTGCTTC | Primers for disrupting *wdlU* |
| *Δ wdlU*-R | CGTAAAGTGTCGGCAATCGAGCGGAAGGTGAAGTCCGTGATTCCGGGGATCCGTCGACC | |
| *Δ wdlU*-YZ-F | GTGGGAGATCTTCGAGGAGT | Primers for *ΔwdlU* construct verification |
| *Δ wdlU*-YZ-R | AGCACGGGATCATCAACGAC | |
| kasOp*-wdlA-ter-F | AATTCGATATCGCGCGCGGCCGCtgttcacattcgaacggtctctgctttgacaacatgctgtgcggtgttgtaaagtcgtggccaggagaatacgacagcgtgcaggactgggggagttCATATGGTGCCGGGGGACAAAGTGGT (*Not*I, *Nde*I) | Primers for pCL08 construction |
| kasOp*-wdlA-ter-R | TCGTTAGTTAGGCTAACTAGTaaaaaaaaccccgccctgtcagggggcggggttttttttctctagtaGGCGGTCAGGCCACGTCCATGGCCG (*Spe*I) | |
| pCL08-YZ-1-F | GACAACATGCTGTGCGGTGT | Primers for pCL08 construct verification |
| pCL08-YZ-1-R | GGGCTCCGGTCGAGTACGTC | |
| kasOp*-wdlB-ter-1-F | AATTCGATATCGCGCGCGGCCGCtgttcacattcgaacggtctctgctttgacaacatgctgtgcggtgttgtaaagtcgtggccaggagaatacgacagcgtgcaggactgggggagttCATATGGTGGGTCACCGTGAGGACTT (*Not*I, *Nde*I) | Primers for pCL09 construction |
| kasOp*-wdlB-ter-1-R | TCGTTAGTTAGGCTAACTAGTaaaaaaaaccccgccctgtcagggggcggggttttttttctctagtaGGCCGTCACTTCACGACGGCCTCGT (*Spe*I) | |
| pCL09-YZ-F | CGATATCGCGCGCGGCCGCT | Primers for pCL09 construct verification |
| pCL09-YZ-R | CGTTAGTTAGGCTAACTAGT | |
| kasOp*-wdlB-ter-2-F | ACCATGCATAGATCTAAGCTTaaaaaaaaccccgccctgtcaggggcggggttttttttctctagtaGGCCGTCACTTCACGACGGCCTCGT (*Hin*dIII) | Primers for pCL10 construction |
| kasOp*-wdlB-ter-2-R | GCGAAAAGCCGAGAACCTAGGtgttcacattcgaacggtctctgctttgacaacatgctgtgcggtgttgtaaagtcgtggccaggagaatacgacagcgtgcaggactgggggagttCATATGGTGGGTCACCGTGAGGACTT (*Avr*II, *Nde*I) | |
| pCL10-YZ-F | ACCATGCATAGATCTAAGCT | Primers for pCL10 construct verification |
| pCL10-YZ-R | ATGGCGAAAAGCCGAGAACC | |

**Table S4.** Strains and plasmids used and constructed in this study.

| Strain or Plasmid | Description | Sources or References |
|---|---|---|
| ***Streptomyces*** | | |
| *S. lividans* GX28 | Host for heterologous expression of BACs | [1] |
| *S. lividans* GX28/vector | *S. lividans* GX28 contains empty vector pMSBBAC1 | This work |
| *S. lividans* GX28 /pBAC-wdl | *S. lividans* GX28 integrated with plasmid pBAC-wdl which contains *wdl* biosynthetic gene cluster | This work |
| *ΔwdlA* | *wdlA* inactivation mutant of *S. lividans* GX28/pBAC-wdl | This work |
| *ΔwdlB* | *wdlB* inactivation mutant of *S. lividans* GX28/pBAC-wdl | This work |
| *ΔwdlF* | *wdlF* inactivation mutant of *S. lividans* GX28/pBAC-wdl | This work |
| *ΔwdlG* | *wdlG* inactivation mutant of *S. lividans* GX28/pBAC-wdl | This work |
| *ΔwdlH* | *wdlH* inactivation mutant of *S. lividans* GX28/pBAC-wdl | This work |
| *ΔwdlO* | *wdlO* inactivation mutant of *S. lividans* GX28/pBAC-wdl | This work |
| *ΔwdlU* | *wdlU* inactivation mutant of *S. lividans* GX28/pBAC-wdl | This work |
| *OwdlA* | Plasmid pCL05 was integrated into the starin *S. lividans* GX28/pBAC-wdl for overexpressing gene *wdlA* | This work |
| *OwdlB* | Plasmid pCL06 was integrated into the starin *S. lividans* GX28/pBAC-wdl for overexpressing gene *wdlB* | This work |
| *OwdlAB* | Plasmid pCL07 was integrated into the starin *S. lividans* GX28/pBAC-wdl for overexpressing gene *wdlA* and gene *wdlB* | This work |
| *ΔwdlO+OwdlA* | Plasmid pCL05 was integrated into the starin *S. lividans* GX28/*ΔwdlO* for overexpressing gene *wdlA* | This work |
| *ΔwdlO+OwdlB* | Plasmid pCL06 was integrated into the starin *S. lividans* GX28/*ΔwdlO* for overexpressing gene *wdlB* | This work |
| *ΔwdlO+OwdlAB* | Plasmid pCL07 was integrated into the starin *S. lividans* GX28/*ΔwdlO* for overexpressing gene *wdlA* and gene *wdlB* | This work |
| ***E. Coli*** | | |
| DH10B | Host strain for cloning | Invitrogen |
| BW25113/pIJ790 | Host strain for λ Red-mediated PCR targeting | [2] |
| DH5α/BT340 | Host strain for in-frame deletion | [2] |
| ET12567 | Host strain of BACs | [3] |
| ET12567/pUB307 | Helper strain for conjugation between *E. coli* and *Streptomyces* | [4] |
| **Plasmids** | | |

(table to be continued)

(table continued)

| | | |
|---|---|---|
| pMS82 | *Hyg*$^{r}$, *oriT*, *int-attP*$^{\varphi BT1}$, integrative plasmid | [5] |
| pJTU6722 | *Ery*$^{r}$, PCR template for *eryB* cassette | [6] |
| pMSBBAC1 | *oriT*, *int-attP*$^{\varphi C31}$, *Apr*$^{r}$, BAC vector | [7] |
| pBAC-wdl | BAC contains the weddellamycin biosynthetic gene cluster | This work |
| pCL01 | *wdlA* was deleted from pBAC-wdl | This work |
| pCL02 | *wdlB* was deleted from pBAC-wdl | This work |
| pCL03 | *wdlF* was deleted from pBAC-wdl | This work |
| pCL04 | *wdlG* was deleted from pBAC-wdl | This work |
| pCL05 | *wdlH* was deleted from pBAC-wdl | This work |
| pCL06 | *wdlO* was deleted from pBAC-wdl | This work |
| pCL07 | *wdlU* was deleted from pBAC-wdl | This work |
| pCL08 | pMS83 was inserted into a *kasO*p*-*wdlA-ter* cassette | This work |
| pCL09 | pMS83 was inserted into a *kasO*p*-*wdlB-ter-1* cassette | This work |
| pCL10 | pMS83 was inserted into a *kasO*p*-*wdlA-ter* cassette and a *kasO*p*-*wdlB-ter-2* cassette | This work |

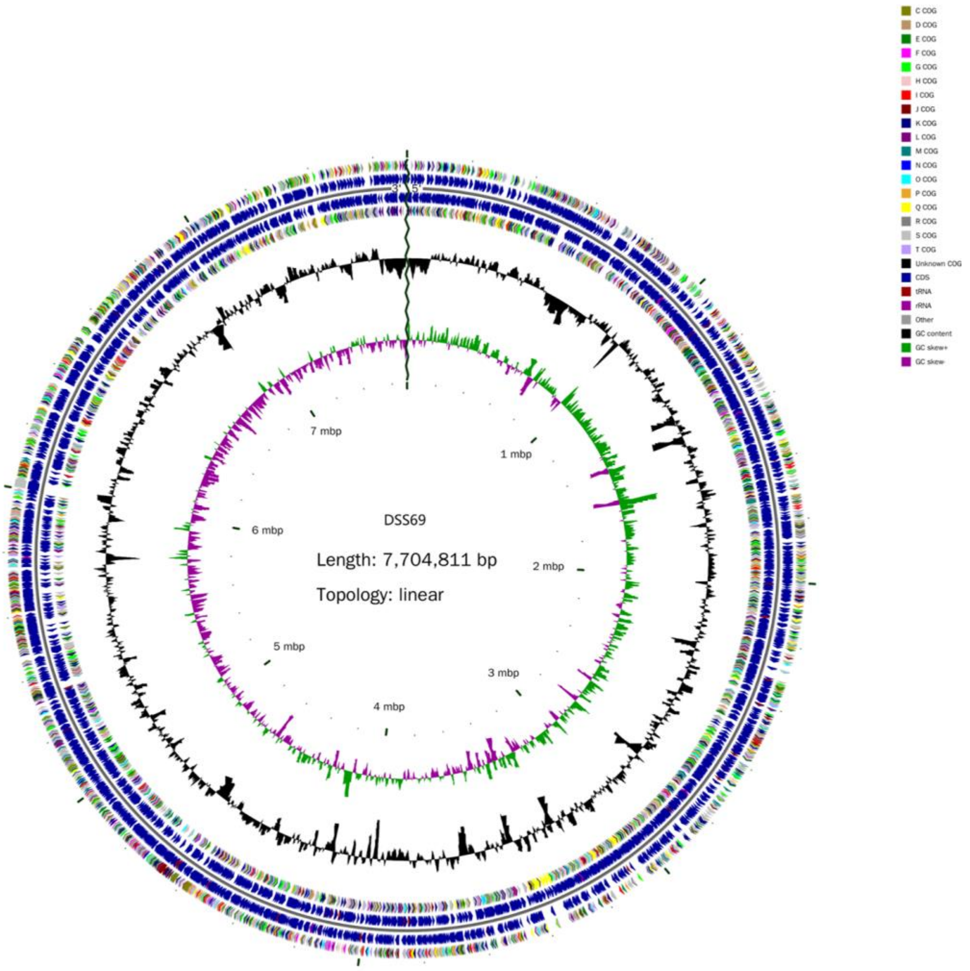


| Feature | Chromosome Characteristics |
|---|---|
| Genome topology | Linear |
| Chromosome size (bp) | 7,704,811 |
| Average GC content (%) | 71.61% |
| Protein-coding genes | 6,689 |
| rRNAs number | 18 |
| tRNAs number | 65 |

**Figure S1.** The complete genome and features of *Streptomyces* sp. DSS69. The seven circles (inner to outer) represent the scale, GC skew, the GC content, the COG to each CDS (4th and 7th circles) and the positions of CDS, tRNA and rRNA on the genome (5th and 6th circles).

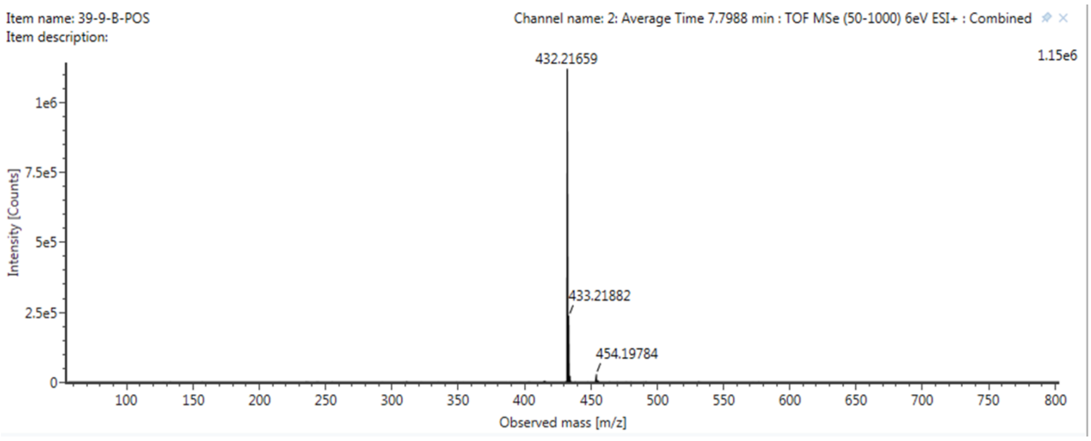


**Figure S2.** HR-ESI-MS spectra of compound **1**.

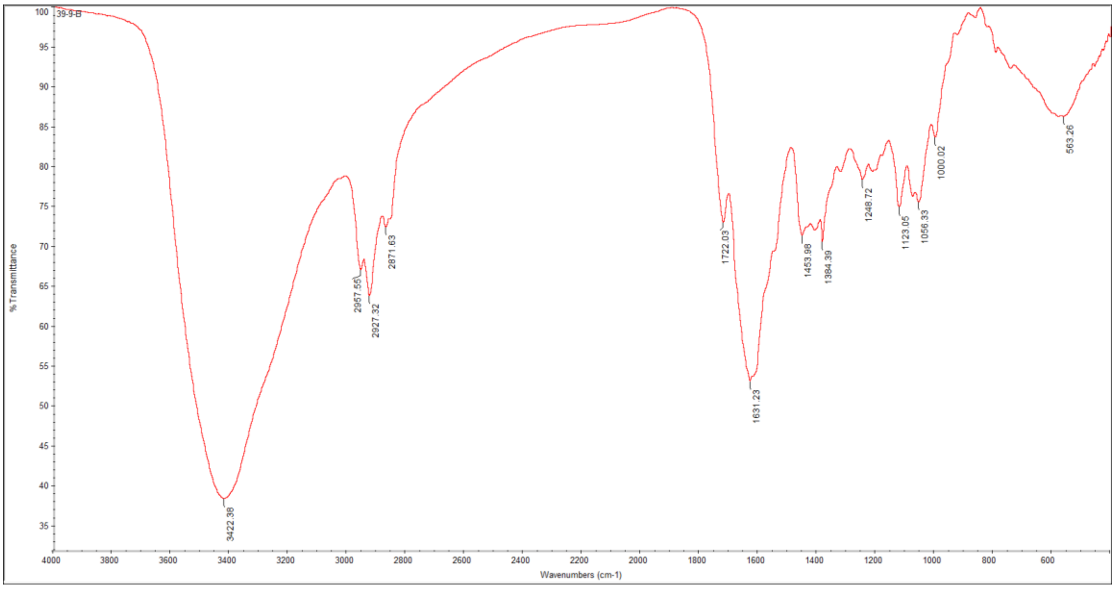


**Figure S3.** IR spectra of compound **1**.

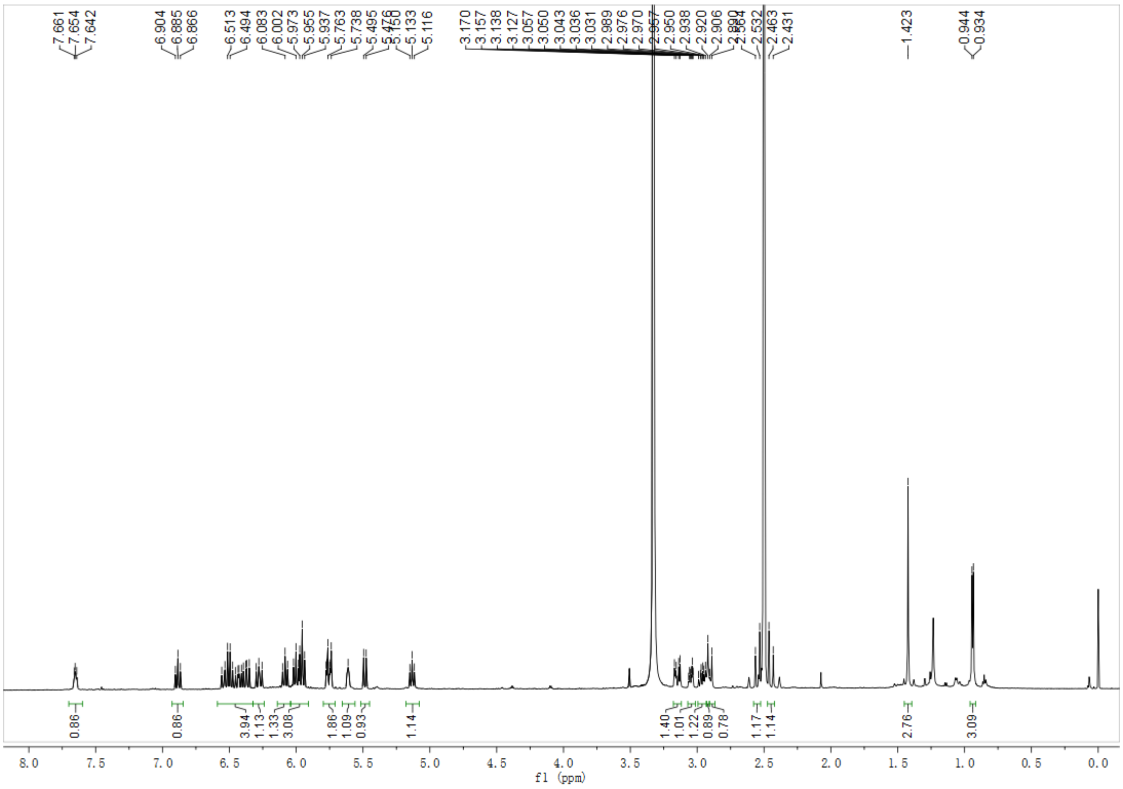

**Figure S4.** $^{1}$H NMR spectrum of compound **1** recorded in DMSO-$d_6$ (600 MHz).

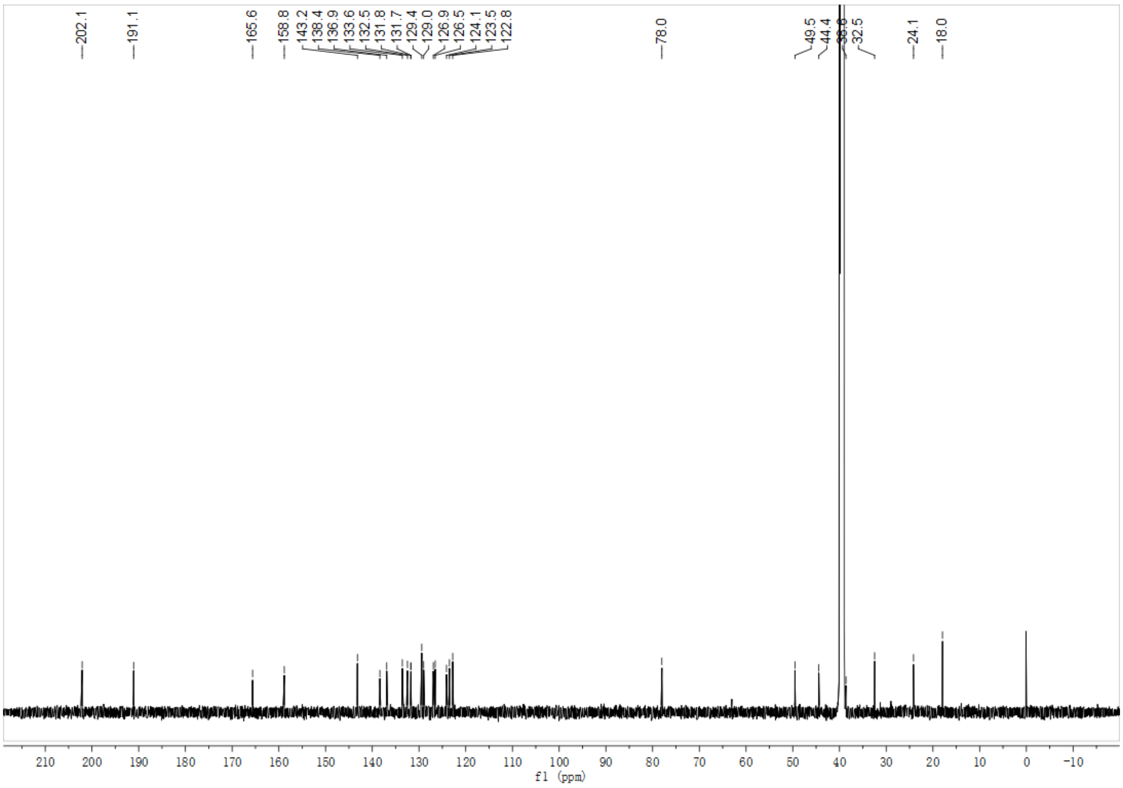

**Figure S5.** $^{13}$C NMR spectrum of compound **1** recorded in DMSO-$d_6$ (150 MHz).

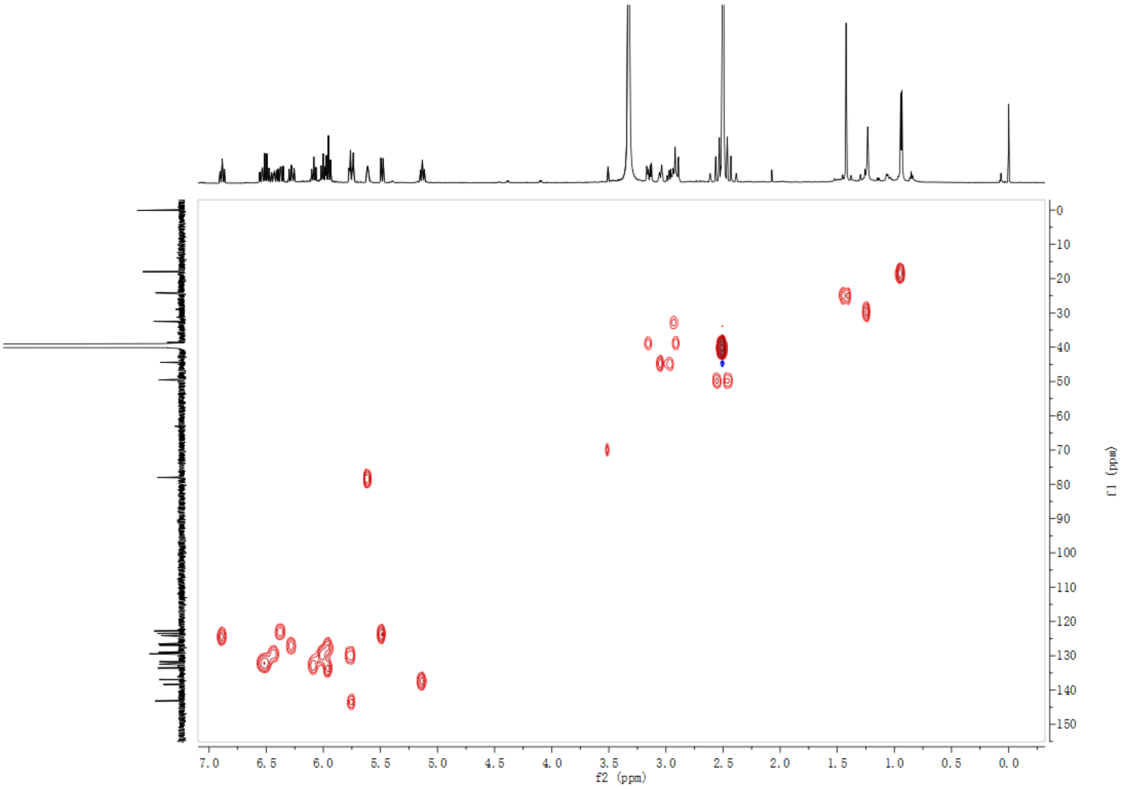


**Figure S6.** HSQC spectrum of compound **1** recorded in DMSO-$d_6$ (600 MHz).

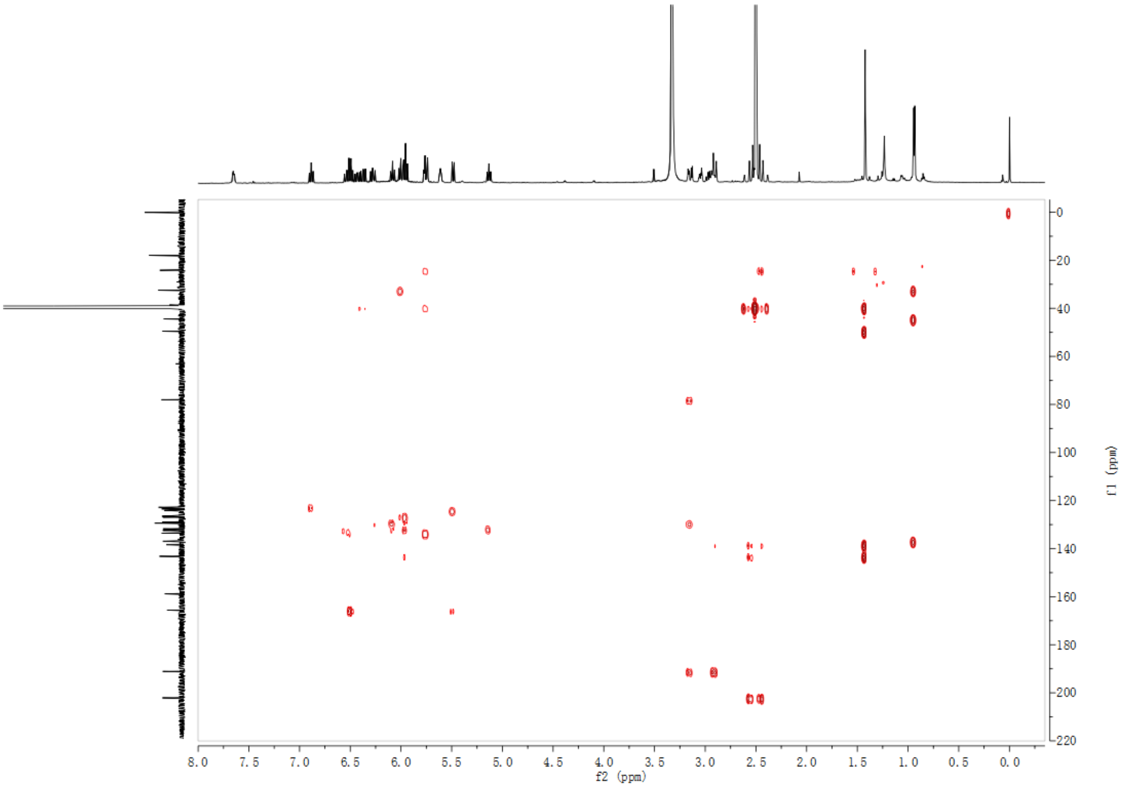


**Figure S7.** HMBC spectrum of compound **1** recorded in DMSO-$d_6$ (600 MHz).

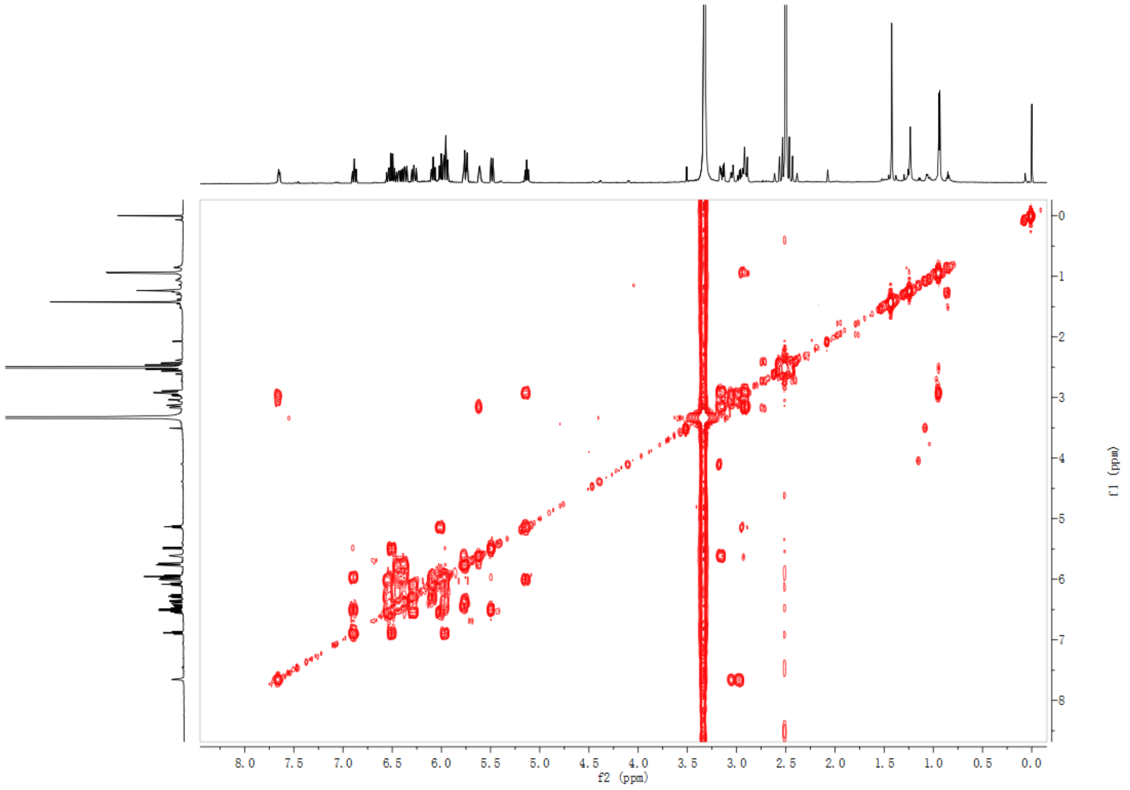


**Figure S8**. $^{1}H$-$^{1}H$ COSY spectrum of compound **1** recorded in DMSO-$d_6$ (600 MHz).

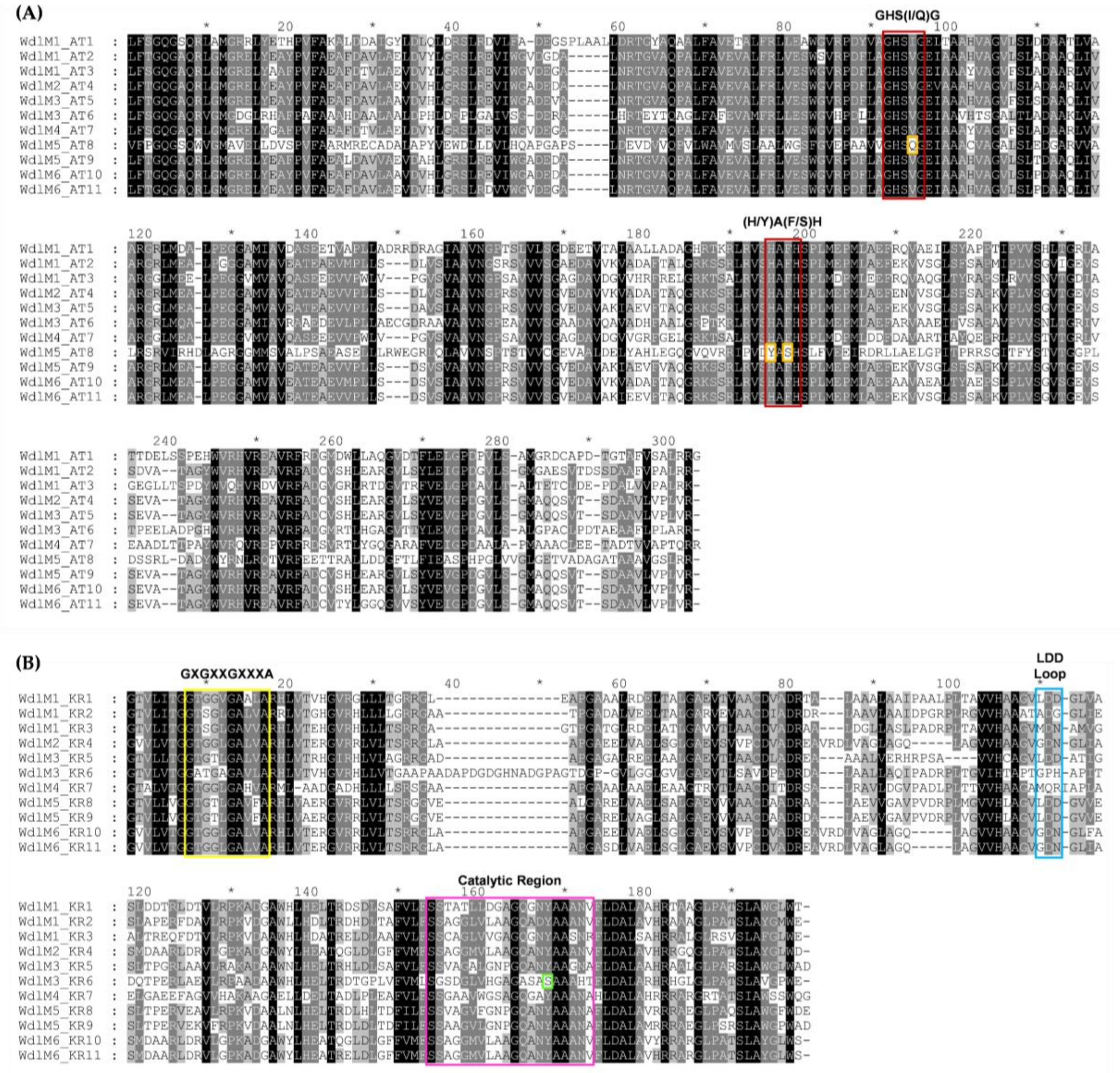


**Figure S9.** Multiple sequence alignment of AT domains and KR domains. **(A)** Alignment of AT domains. Red boxes indicate the characteristic residues for substrate recognition. AT domains in module 8 is predicted to be methylmalonyl-CoA-specific AT, which possess GHSQG and YASH motifs that are distinctly different with other malonyl-CoA specific ATs (indicated in yellow boxes). **(B)** Yellow box indicates conserved motif for an NADP(H) binding site. Blue box indicates LDD motif. The catalytic region is indicates as pink box. The KR domain in module 6 is predicted to be the C1 subtype, which laks the Y motif (indicated in green box).

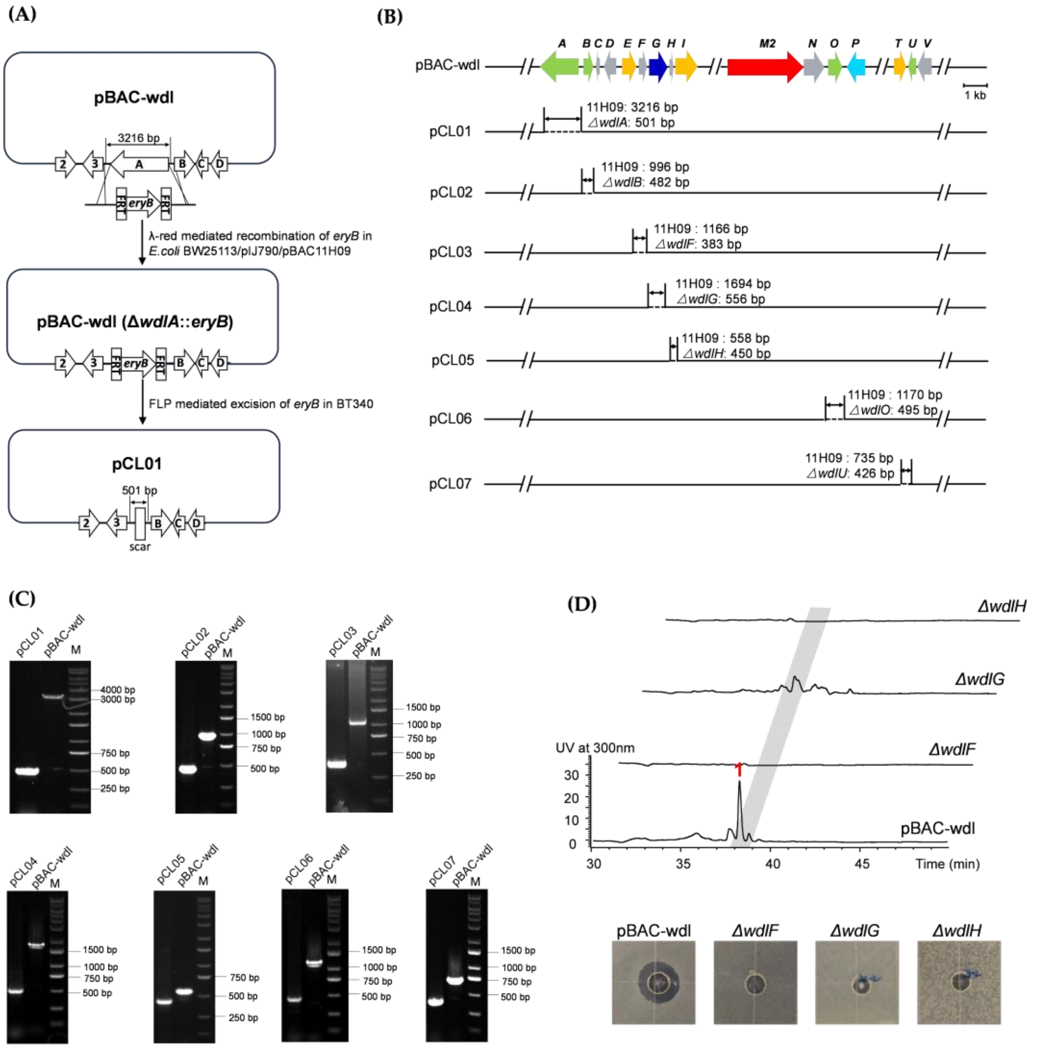


**Figure S10.** Disruptions of weddellamycin biosynthetic genes via PCR-targeting and PCR verification. **(A)** Schematic representation for inactivation of *wdlA* as an example. **(B)** Genetic map showing the *wdl* biosynthetic gene cluster (pBAC-wdl) and the disrupted regions for all mutants. Deleted regions are shown with dotted lines. PCR amplifications were designed for confirming each plasmid, and the amplified regions are shown as double-headed arrows with the expected sizes shown for the pBAC-wdl and mutants. **(C)** PCR vertification of the gene deletion mutants compared to original one pBAC-wdl. M is the 1 kb Plus DNA Ladder (GenStar) and numbers next to the gels are sizes of the indicated bands in bp. (D) HPLC profiles of *S. lividans GX28*/pBAC-wdl and the gene deletion mutants *ΔwdlF*, *ΔwdlG* and *ΔwdlH*. Crude extract (20 μL) was added to the central wells in the agar plates premixed with *B. altitudinis* as an indicator. Biological activity was indicated by the zones of growth inhibition after 24 h of incubation at 37℃.

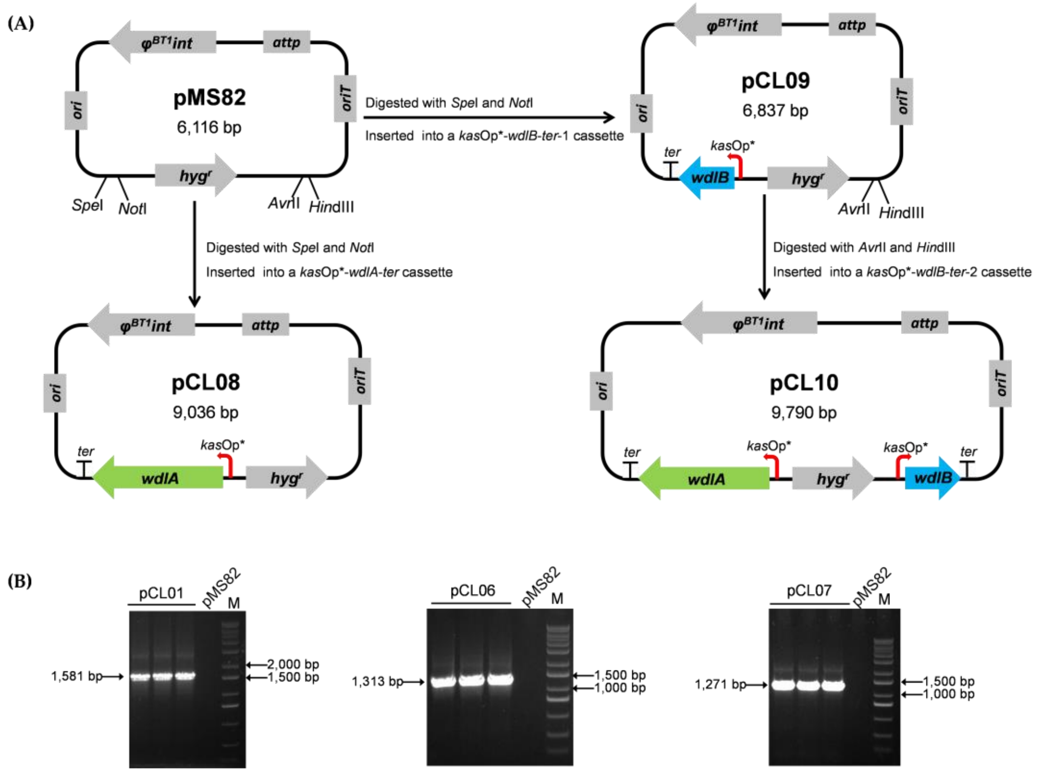


**Figure S11.** Schematic maps of overexpression plasmids and PCR verification. **(A)** After linearizing the pMS82 plasmid using *Not*I and *Spe*I restriction sites, the fragments *kas*Op*-*wdlA*-*ter* and *kas*Op*-*wdlB*-*ter*-1 were inserted into the plasmid using a one-step cloning method, resulting in overexpression plasmids pCL08 and pCL09. The plasmid pCL10 was obtained by linearizing pCL08 through *Avr*II and *Hin*dIII double digestion, and then inserting the fragment of *kas*Op*-*wdlB*-*ter*-2 using the same one-step cloning method. **(B)** PCR vertification of the overexpression plasmids compared to original one pMS82. M is the 1 kb Plus DNA Ladder (GenStar) and numbers next to the gels are sizes of the indicated bands in bp.

## References


1. Peng, Q.; Gao, G.; Lü, J.; Long, Q.; Chen, X.; Zhang, F.; Xu, M.; Liu, K.; Wang, Y.; Deng, Z.; Li, Z.; Tao, M. Engineered *Streptomyces lividans* Strains for Optimal Identification and Expression of Cryptic Biosynthetic Gene Clusters. *Front. Microbiol.* **2018**, *9*.
2. Datsenko, K.A.; Wanner, B.L. One-Step Inactivation of Chromosomal Genes in *Escherichia coli* K-12 Using PCR Products. *Proceedings of the National Academy of Sciences - PNAS* **2000**, *97*, 6640-6645.
3. Douglas J. MacNeil; Keith M. Gewain; Carolyn L. Ruby; Gabe Dezeny; Patriee H. Gibbons; Maeneil, T. Analysis of *Streptomyces avermitilis* Genes Required for Avermectin Biosynthesis Utilizing a Novel Intergration Vector. *Gene* **1992**, *1*, 61-68.
4. Fiona Flett; Vassilios Mersinias; Smith, C.P. High Efficiency Intergeneric Conjugal Transfer of Plasmid DNA from *Escherichia coli* to Methyl DNA-restricting *Streptomycetes*. *FEMS Microbiol. Lett.* **1997**, 223-229.
5. Gregory, M.A.; Till, R.; Smith, M.C.M. Integration Site for *Streptomyces* Phage φBT1 and Development of Site-Specific Integrating Vectors. *J. Bacteriol.* **2003**, *185*, 5320.
6. Gao, G.; Liu, X.; Xu, M.; Wang, Y.; Zhang, F.; Xu, L.; Lv, J.; Long, Q.; Kang, Q.; Ou, H.; Wang, Y.; Rohr, J.; Deng, Z.; Jiang, M.; Lin, S.; Tao, M. Formation of an Angular Aromatic Polyketide from a Linear Anthrene Precursor via Oxidative Rearrangement. *Cell Chemical Biology* **2017**, *24*, 881-891.
7. Huang, S.; Li, N.; Zhou, J.; He, J. Construction of a New Bacterial Artificial Chromosome (BAC) Vector for Cloning of Large DNA Fragments and Heterologous Expression in *Streptom*yces. *Acta Microbiologica Sinica* **2012**, *52*, 30-37.
